# Supplementary material for: A novel mitochondrial complex I ROS inhibitor partially improves muscle regeneration in adult but not old mice
Source: Redox Biol. 2023 Jun 2;64:102770. doi: 10.1016/j.redox.2023.102770 (PMC10267642; doi:10.1016/j.redox.2023.102770)
Supplement: Multimedia component 1 [file mmc1.docx]

**A Novel Mitochondrial Complex I ROS Inhibitor Partially Improves Muscle Regeneration in Adult but not Old Mice**

Gavin Pharaoh^1^*, Ethan L. Ostrom^1^*, Rudy Stuppard^1^, Matthew Campbell^1^, Jens Markus Borghardt^2^, Michael Franti^3^, Antonio Filareto^3^, David J. Marcinek^1,4^

^1^Department of Radiology, University of Washington School of Medicine

^2^Research DMPK, Department of Drug Discovery Sciences, Boehringer Ingelheim Pharma GmbH & Co. KG, Biberach, Germany

^3^Research Beyond Borders, Boehringer Ingelheim Pharmaceuticals, Ridgefield, Connecticut, USA.

^4^Institute for Stem Cell and Regenerative Medicine, University of Washington School of Medicine.

*contributed equally to this report

**Abstract**

It is unclear whether mitochondrial dysfunction and redox stress contribute to impaired age-related muscle regenerative capacity. Here we characterized a novel compound, BI4500, that inhibits the release of reactive oxygen species (ROS) from the quinone site in mitochondrial complex I (site I_Q_). We tested the hypothesis that ROS release from site I_Q_ contributes to impaired regenerative capacity in aging muscle. Electron transfer system site-specific ROS production was measured in adult and aged mouse isolated muscle mitochondria and permeabilized gastrocnemius fibers. BI4500 inhibited ROS production from site I_Q_ in a concentration-dependent manner (IC_50_ = ~985 nM) by inhibiting ROS release without impairing complex I-linked respiration. *In vivo* BI4500 treatment decreased ROS production from site I_Q_. Muscle injury and sham injury were induced using barium chloride or vehicle injection to the tibialis anterior (TA) muscle in adult and aged male mice. On the same day as injury, mice began a daily gavage of 30 mg/kg BI4500 (BI) or placebo (PLA). Muscle regeneration (H&E, Sirius Red, Pax7) was measured at 5 and 35 days after injury. Muscle injury increased centrally nucleated fibers (CNFs) and fibrosis with no treatment or age effect. There was a significant age by treatment interaction for CNFs at 5- and 35-days post injury with significantly more CNFs in BI adults compared to PLA adults. Muscle fiber cross-sectional area (CSA) recovered significantly more in adult BI mice (-89±365 µm^2^) compared to old PLA (-599±153 µm^2^) and old BI (-535±222 µm^2^, mean±SD). *In situ* TA force recovery was measured 35 days after injury and was not significantly different by age or treatment. Inhibition of site I_Q_ ROS partially improves muscle regeneration in adult but not old muscle demonstrating a role for CI ROS in the response to muscle injury. Site I_Q_ ROS does not contribute to impaired regenerative capacity in aging.

**Keywords: Reverse Electron Transport (RET), Muscle Injury and Regeneration, Mitochondrial Reactive Oxygen Species (ROS), Barium Chloride Injury, Sarcopenia**

**Highlights**

- BI4500 decreases ROS production from site I_Q_ of the mitochondrial ETS
- BI4500 does not significantly impair CI-linked mitochondrial respiration.
- 8-week feeding of BI4500 decreases site I_Q_ ROS from adult and old mice.
- BI4500 treatment increases muscle CNFs and improves CSA recover after injury.
- Muscle function is similar between BI4500 treatment or placebo 35 days post-injury.

**Introduction**

Skeletal muscle is a highly plastic tissue, capable of adapting to various stressors encountered throughout life[1, 2]. Aging is associated with declines in adaptive muscle plasticity related to muscle function and mass[3-5], which exacerbate muscle loss, low quality of life and increased risk of morbidity and mortality[6]. Maintaining muscle mass and function are therefore critical for maintaining independence and quality of life in older adults. Muscle satellite cells (MuSCs) are tissue-resident stem cells responsible for the regeneration of new muscle through gene programs that regulate proliferation, differentiation and fusion of new myotubes[7]. These gene programs are turned on in response to various models of muscle injury, as well as exercise[8]. However, aging leads to MuSC senescence preventing activation of the proliferation program, decreasing regenerative capacity, and increasing susceptibility to age related comorbidities and dysfunction[9].

One potential explanation for the declining repair capacity of skeletal muscle satellite cells is age-associated mitochondrial dysfunction and concomitant redox stress[10]. Aging mitochondria have increased reactive oxygen species (ROS) production, shifting the cellular milieu to a more oxidized state, which could alter satellite cell activation and contribute to declining regenerative capacity[10, 11]. Several studies have demonstrated that targeting redox active compounds to mitochondria or genetic over expression of antioxidant enzymes in mitochondria can restore age-related declines in function and the redox proteome to young levels[12-14]. Therefore, targeting mitochondria has therapeutic potential for developing and testing new pharmaceuticals to improve age-related muscle plasticity and regeneration.

We previously reported mitochondrial electron transport system (ETS) complex I protein abundance and function are affected by aging in mouse skeletal muscle[15]. ROS released from complex I sites I_F_ and I_Q_ in skeletal muscle mitochondria account for about half of the total ROS production during rest and the vast majority of mitochondrial ROS during exercise[16]. ROS produced from these sites by reverse electron transport (RET) drives tissue damage after ischemia-reperfusion injury and during aging, and limiting ROS released from complex I has become a major research topic to improve lifespan and healthspan [17-19]. The complex relationship between CI ROS production and muscle differentiation has been studied *in vitro*, but it is unknown how CI ROS production contributes to muscle differentiation *in vivo* [20-23]. Here we characterized a novel compound (BI4500) that inhibits superoxide (O_2_^−^) release from RET at mitochondrial complex I_Q_ (**Graphical Abstract**). We then used this compound to test the hypothesis that treatment with a RET complex I inhibitor would improve muscle regeneration after injury in adult and old mice compared to placebo.

**Materials and Methods**

*Animal Husbandry*.

C57Bl6/J male and female mice were purchased from The Jackson Laboratory and housed at the University of Washington. All mice were maintained at 21 °C on a 14/10 light/dark cycle at at 30-70% humidity and given standard mouse chow (LabDiet PicoLab® Rodent Diet 20) and water ad libitum unless otherwise specified. This study was reviewed and approved by the University of Washington Institutional Animal Care and Use Committee (IACUC).

*BI4500*.

BI4500 is an indolinone composed of C_20_H_27_N_3_O_3_ and was manufactured by Boehringer Ingelheim International with greater than 99.5% purity.

*Selectivity Study*.

BI4500 was tested for selectivity against 17 enzymes by Boehringer Ingelheim. BI4500 showed greater than 75% inhibition of arachidonate 5-lipoxygenase (ALOX5) and arachidonate 15-lipoxygenase (ALOX15) at 10 µM.

*Direct Superoxide Dismutase Activity Assay*.

To test whether BI4500 had direct O_2_^−^ scavenging capabilities, superoxide dismutase (SOD) activity was determined using the Cayman Superoxide Dismutase Assay Kit (Cayman Chemical Item No. 706002, MI, USA) according to kit instructions without biological samples. Detailed methods are available in the supplemental information.

*Direct H_2_O_2_ Scavenging Assay.*

To test whether BI4500 had direct H_2_O_2_ scavenging capabilities, a plate-based Amplex UltraRed assay was used identical to the site-specific isolated mitochondria ROS assay but without biological samples. BI concentrations of 0, 1, and 10 µM were incubated with a standard curve of H_2_O_2_ in triplicate.

*Mitochondrial Isolation and Superoxide Release from Sites in Complex I.*

The gastrocnemius, quadriceps femoris, and tibialis anterior (TA) muscles were dissected from both hindlimbs, and mitochondrial isolation was performed by differential centrifugation. The production of O_2_^−^ and hydrogen peroxide (H_2_O_2_) from the ETS complex I sites was measured using Amplex UltraRed (Molecular Probes, Eugene, OR) as described in previous publications with some modifications[24, 25]. ROS production from site I_Q_ reverse was measured in mitochondria from 12 mo adult and 24-26 mo old female mice using 5 mM succinate and 0.3 μM S3QEL1.2 normalized to the inhibitor control of 5 mM succinate, 0.3 uM S3QEL1.2, and 1 uM nigericin at the same concentrations of BI4500 in duplicate (**Figure 1A**). Succinate as a substrate stimulates O_2_^−^ release from sites I_Q_, I_F_, and III_Qo_ [26]. S3QEL1.2 (Sigma SML1554) was added to specifically inhibit site III_Qo_. Nigericin (Sigma N7143) collapses the pH gradient necessary for I_Q_ O_2_^−^ release. After 8 weeks of control chow or BI4500 chow feeding, ROS production from specific sites was measured in 11-12 mo adult and 24-26 mo old female mice using the substrate and inhibitors described in columns in **Table S1** in triplicate (**Figure 1C**). Detailed methods are available in the supplemental information.

*Muscle Fiber Preparation and Respirometry and Fluorometry.*

Several permeabilized red gastrocnemius muscle fiber bundles per animal were prepared from 6 mo adult and 29 mo old female mice as previously described with some modifications (**Figure 1B**) [27-29]. Fibers were permeabilized and washed with vehicle, 1, or 10 µM BI4500. To measure site-specific hydroperoxide production in permeabilized muscle fibers stimulated with succinate, we developed a SUIT protocol designed to isolate ROS production from specific sites using sequential additions of succinate, S3QEL1.2, nigericin, and ADP. Detailed methods were previously described, and additional details are available in the supplemental information[27-29].

*BI Chow Feeding and Oral Gavage Pharmacokinetic Studies.*

We fed adult (9-10 mo) and aged (22-24 mo) female mice control chow for 2 weeks then BI4500 chow (580 mg BI4500 per kg chow) for 8 weeks (**Figure S1A, 1C**). Age- and sex- matched control mice were maintained on control chow. The mice were sacrificed at 12 months and 24-26 months of age. Plasma was collected to measured BI45000 concentration and mitochondria were isolated from the muscles of these mice and used to measure ROS production. ROS production was compared to age- and sex-matched mouse fed standard chow. A single dose and once-daily oral gavage pharmacokinetic studies at 30 mg/kg BI4500 were performed (**Figure 1D**). Additional details are available in supplemental methods. BI4500 concentrations were determined using mass spectrometry by PharmaCadence Analytical Services (Hatfield, Pennsylvania).

*Muscle Injury and Regeneration Study with BI4500 Oral Gavage.*

We used intramuscular sham and barium chloride (BaCl_2_) injections to injure TA muscles of adult (9-11mo) and old (26mo) animals **(Figure 1E**)**.** Animals began daily oral gavage treatment with either placebo control (PLA) or 30mg/kg/day BI4500 compound (BI) the same day as muscle injury. Treatment was administered daily for either 5 days or 35 days and the TA muscle was collected. The cohort that was treated for 35 days underwent *in situ* muscle function testing followed by TA collection for histological measures. Additional details are available in supplemental methods.

*In Situ TA Muscle Function*.

On day 35, animals were anesthetized with 4% isoflurane induction and placed on a heat pad under 1-2% isoflurane to maintain anesthesia during the procedure. Skin was removed from the ankle to the knee, followed by careful dissection of the surrounding connective tissue superficial to the TA. The distal TA tendon was cut below the ankle and sutured to the force transducer. Electrodes were placed subcutaneously behind the knee to stimulate the peroneal nerve. Following electrode placement, a voltage titration was performed to identify maximal twitch force (6-10V) followed by a force frequency test. Force was measured every minute with increasing frequency from 10 to 200 hertz. Maximal TA force was recorded from the force frequency curve. Both TA muscles were tested in each animal with the injured limb always being tested first to control for an order effect of muscle stimulation.

*Histology.*

After TA muscle collection, muscles were immediately placed in 0.4% paraformaldehyde – PBS solution for 2 hours at 4ºC followed by incubation in 20% sucrose-PBS at 4ºC overnight. Muscles were removed from sucrose-PBS and immediately frozen in OCT using liquid N_2_ cooled isopentane. Frozen tissue was stored at -80ºC until sectioning. Eight µm cross sections were cut using a Leica CM1950 (Leica Biosystems, Deer Park IL) and placed on glass slides. Slides were stored at -80ºC until staining.

*Staining & Imaging*.

H&E, Sirius red, or immunohistochemistry protocols were used to characterize muscle morphology, centrally nucleated fibers, fibrosis, and Pax7 positive cells, respectively, using the protocols previously described[30]. Detailed methods are available in the supplemental information.

*Statistical Analysis***.**

Graphing and statistical analysis were performed using GraphPad Prism for OS X (GraphPad Software, San Diego, California USA). For all statistical tests, alpha levels were set to p<0.05. Detailed methods are available in the supplemental information.

**Results**

*BI4500 Inhibits ROS Production from Site I_Q_ in a Dose-Dependent Manner.*

We tested whether BI4500 prevents ROS release from site I_Q_ in muscle mitochondria from adult and old mice with a dose-response of BI4500 (**Figure 1A**). BI4500 inhibits ROS release from site I_Q_ in both adult and aged isolated muscle mitochondria with an IC_50_ of ~985 nM and complete inhibition in aged mitochondria around 5-10 µM (**Figure 2**).

We measured site-specific ROS production in gastrocnemius muscle fibers from adult (6-8 mo) and aged (29 mo) mice after pre-treatment *ex vivo* during the fiber permeabilization and wash steps with vehicle or 1 μM BI4500 (**Figure 1B**). Incubation with vehicle or BI4500 during fiber permeabilization and washing results in ~1 hour of treatment before measuring ROS production. This dose showed inhibitory effects on site I_Q_ ROS production in isolated mitochondria, while avoiding the potential for interference observed at 10 μM in the Amplex Red reaction (**Figure S2E-F**). BI4500 significantly decreased total ROS production with succinate (p<0.05 BI4500 effect) (**Figure 3A)**. In muscle fibers from aged mice, BI4500 decreased ROS production from site I_Q_ (p<0.05 by t-test) (**Figure 3A)**. Site I_F_ was unaffected by BI4500 treatment (**Figure 3A)**. Interestingly, BI4500 pre-treatment decreased the elevated basal hydroperoxide production observed in aged fibers (p<0.05 age effect, p<0.01 BI4500 x age effect), which has previously been correlated with the extent of muscle atrophy in various models including aging (**Figure 3A)**[31]. This is likely due to micromolar doses of BI4500 displaying inhibitory effects on lipoxygenases. A selectivity study of the compound showed greater than 75% inhibition of ALOX5 and ALOX15 lipoxygenases at 10 μM. ALOX15 partially accounts for increased basal hydroperoxides in aging after spontaneous loss of neuromuscular innervation[31]. BI4500 pre-treatment increased the efficiency of electron transport resulting in less ROS produced per oxygen consumed (p<0.05 BI4500 effect) **(Figure 3B**).

*Ex Vivo Treatment with BI4500 Does Not Significantly Impair Mitochondrial Respiration in Permeabilized Muscle Fibers.*

We measured mitochondrial respiration in permeabilized gastrocnemius muscle fibers from adult (6-8 mo) and aged (29 mo) mice pre-treated *ex vivo* with vehicle, 1 μM, or 10 μM BI4500 during the fiber permeabilization and wash steps to identify if the compound inhibits mitochondrial respiration (**Figure 1B, 3C).** Pre-treatment with BI4500 did not significantly decrease respiration capacities under any substrate condition in either age group (**Figures3C**). Respiration capacities were all significantly decreased with age (p<0.05 age effect).

*BI4500 Has Low Direct Superoxide and H_2_O_2_ Scavenging.*

We determined whether BI4500 directly scavenges O_2_^−^ and H_2_O_2_ in assay conditions without any biological samples. BI4500 has SOD activity of 0.035 units per nmole of compound (**Figure S2A-B**). Therefore, in the Amplex UltraRed assay conditions there is approximately 0.35 U/ml of SOD activity in the highest concentration (10 μM) of BI4500 (**Figure S2A-B**). However, the buffer has 25 U/ml of SOD in the plate-based assay buffer, so BI4500 SOD activity represents a small fraction of the total SOD activity of the assay buffer (1.4% at 10 μM BI4500). We also measured the H_2_O_2_ scavenging properties of several doses of BI4500 in assay conditions without any biological samples. There was no difference in the H_2_O_2_ standard curve at 1 μM BI4500 in our plate-based assay or O2k assays compared to vehicle control, while 10 μM BI4500 decreased the fluorescence at each H_2_O_2_ concentration in the O2k (**Figure S2C-F**). However, we determined the IC_50_ to be less than 1 μM, therefore the effects observed on assay conditions at 10 μM BI4500 are unlikely to explain the inhibition of site I_Q_ at lower concentrations. Thus, the inhibition of O_2_^−^ release we observe from site I_Q_ after BI4500 treatment is not due to direct scavenging of the O_2_^−^ but by inhibition of O_2_^−^ release.

*Eight Weeks of BI Chow Feeding Decreases ROS Production from Site I_Q_ in Isolated Mitochondria***.**

We fed adult (9-10 mo) and aged (22-24 mo) female mice BI4500 chow for 8 weeks (**Figure 1C**). There was no difference in food consumption after switching to BI chow (**Figure S1A**). Unfortunately, concentrations of BI4500 were below the limit of quantitation for the plasma samples. Mitochondria were isolated from the combined hindlimb muscles (gastrocnemius, quadriceps, and TA) of BI chow fed mice as well as from age- and sex-matched mice fed control chow to measure site-specific ROS production **(Table S1**)**.** Despite the low plasma PK values, animals fed BI chow had significantly lower ROS production from site I_Q_ compared to controls (p<0.0001 BI4500 effect), while site I_F_ reverse was higher (p<0.05 age effect, p<0.0001 BI4500 effect) and site I_F_ forward was unchanged (**Figure 4A**). Total combined ROS produced by sites I_F_ and I_Q_ were similar across ages and treatments (**Figure 4B**). 8-week consumption of BI4500 chow did not significantly affect body mass or hindlimb muscle masses (**Figure 4C-D).**

*Contribution of I_Q_ ROS Production to Muscle Regeneration*.

Following these mechanistic studies, we wanted to test the role of complex I_Q_ on muscle regeneration after injury in adult and aged mice. We hypothesized that BI4500 (BI) treatment would improve muscle fiber regeneration in aged animals compared to placebo control (PLA).

To test muscle regeneration, we performed barium chloride (BaCl_2_) injury on one TA muscle and sham injury on the contralateral TA muscle of adult and aged male mice and simultaneously began a daily oral gavage treatment with BI4500 (**Figure 1E**). We changed to oral gavage administration, since compound concentrations in plasma following chow treatment were below detectable limits. Plasma concentrations of BI4500 after one week of 30 mg/kg once daily oral gavage were 18.4 ± 8.2 µM (n = 7, 1 h post dose, mean ± SD), which was in range of the observed Cmax in previous PK studies after oral dosing (**Figure 1D**).

*Muscle Regeneration Five Days Post-Injury*.

Five days after muscle injury there was a significant increase in the number of CNFs of all injured limbs compared to sham controls illustrated in **Figure 5A**. There was a significant interaction effect between age and BI4500 treatment (p=0.045 by Two-Way ANOVA) for the increase in CNFs (∆) in the injured versus sham limbs (**Figure 5A**). Specifically, there was a larger increase in CNFs in the injured limb compared to sham in adult animals treated with BI compared to adult animals treated with PLA (p=0.043 by Tukey’s post-hoc test), while there was no significant difference between the increase in CNFs of the injured limb in PLA and BI treated old animals. Additional quantification of H&E was not performed at 5 days due to the severe muscle damage and clear necrosis at this timepoint. Picrosirius red staining was used to assess muscle fibrosis. Baseline tissue fibrosis was elevated in the sham muscles from old animals consistent with previous reports (**Figure 5B**) [32, 33]. BaCl_2_ injection significantly increased fibrosis in all groups five days after injury (**Figure 5B)**. There were no significant differences in the increased (∆) fibrosis response to injury with age or BI4500 treatment among the groups (**Figure 5B**). Pax7 staining for satellite cell abundance in response to muscle injury shows no significant differences between adult and old animals or any BI4500 effect (**Figure 5C**). This is likely due to the large variation in satellite cell proliferation in response to injury.

*Muscle Regeneration 35 Days Post-Injury*.

Muscle cross sections were analyzed by H&E staining 35 days after injury to assess muscle morphology and regeneration. CNFs remained elevated in the injured limbs even 35 days after injury in all groups (**Figure 6A**), suggesting the injured muscle was not fully recovered from the BaCl_2_ injections. There was a main effect of age on CNFs (p<0.001 age effect), with adult mice showing a greater percent increase of CNFs than old mice (**Figure 6A**). There was also a BI4500 treatment effect (p=0.005) and a BI4500 x age interaction (p=0.016). The age by treatment interaction effects were driven by the adult BI treated group, which had a significantly higher increase in CNFs than each of the other groups (**Figure 6A**). These results suggest that BI4500 increased CNFs in adult but not old animals 35 days post injury.

Thirty-five days after muscle injury, muscle fiber cross sectional area (CSA) was still lower in injured limbs compared to sham except for BI treated adult animals (**Figure 6B-C**). CSA showed significant effects of age (p=0.014) which was expected given that generally aging is associated with lower muscle mass and impaired recovery capacity from injury. There was also a BI4500 x age interaction (p=0.029) but no main effect of treatment on CSA. Significantly higher CSA differences in BI adults compared to old PLA (p=0.024) and old BI animals (p=0.049) indicate that the age by treatment interaction was being driven by the adult BI treated animals.

TA muscle fibrosis remained elevated 35 days after injury compared to sham (**Figure 7A** paired comparisons). However, there were no significant differences in the increase in fibrosis between groups in response to injury (**Figure 7A**). Pax7 positive staining showed no major age or treatment differences in response to the muscle injury thirty-five days after injury (**Figure 7B**). Finally, there were no significant differences in maximal TA muscle force recovery by age or treatment group (**Figure 8A-B**).

**Discussion**

Here we characterized a novel compound targeted to mitochondria. BI4500 decreases ROS production from ETS site I_Q_ not by direct ROS scavenging but by inhibiting ROS release (**Graphical Abstract**). The conclusion that the BI compound specifically inhibits ROS production from site I_Q_ and is not a general complex I inhibitor is supported by the absence of any effect of acute *in vitro* treatment on forward or reverse ROS production from the I_F_ site. In contrast to the *in vitro* results, the inhibition of I_Q_ RET ROS production with 8 weeks of *in vivo* treatment with BI4500 was associated with a significant elevation of I_F_ RET oxidant production. These results suggest the *in vivo* treatment resulted in altered structure and function of complex I and support an important signaling role for mitochondrial oxidant production in determining ETS function. Unlike classical site I_Q_ inhibitors like rotenone, BI4500 inhibits I_Q_ ROS release without inhibiting complex I-linked respiration.

It has previously been established that other compounds that inhibit ROS release from site I_Q_ during RET without impairing forward electron flow during respiration do so by binding away from the quinone pocket and modulating the quinone-redox reaction through structural changes in the binding pocket [34]. Conversely, quinone-site inhibitors like rotenone bind directly in the site I_Q_ binding pocket and increase ROS production from site I_F_ by modifying the oxidation-reduction state of Flavin mononucleotide (FMN) [34, 35]. The binding site of BI4500 to complex I is currently unknown, but it is possible that the increase in ROS production at site I_F_ during RET could occur either through modifying the oxidation-reduction state of FMN or by inducing structural changes in complex I.

ROS release from site I_Q_ by RET contributes to both physiological and pathological processes in mammals. RET occurs during aging in flies, and treatment with RET inhibitors extends lifespan and healthspan [19]. RET occurs in several tissues *in vivo* after ischemia-reperfusion injury due to the buildup of succinate[17]. During reperfusion, the resupply of oxygen and abundance of succinate causes high ROS production from RET that results in tissue damage, and treatments that decrease RET O_2_^−^ release improve response to ischemia-reperfusion injury[18]. Supporting the contribution of CI ROS production to cellular damage *in vivo*, oocytes maintain cellular viability and fitness for decades by complete elimination of active complex I, which practically eliminates mitochondrial ROS production[36].

The relationship between CI ROS production and muscle stem cell differentiation is complex, and the role of CI ROS production in muscle differentiation and regeneration *in vivo* is unknown. Increased CI ROS production occurs during muscle myoblast differentiation [20]. Differentiating myoblasts of two different cell lineages require mitochondrial O_2_^−^ production and inhibiting the mitochondrial ROS production with treatment of mitochondrially targeted catalase inhibited muscle differentiation. Further experiments were able to delineate that CI RET specifically was responsible for the redox signaling driving muscle differentiation[23]. Conversely, increased ROS production can also impair differentiation [22]. It has been proposed that ROS production tightly controls muscle stem cell differentiation with a modest increase inducing differentiation but a large increase impairing differentiation (**Graphical Abstract**) [21].

Barium chloride (BaCl_2_) is an established injury model for studying impaired regenerative capacity of aged muscle [37]. By using BI4500 and BaCl_2_ injections, we were able to probe the role of I_Q_ ROS in muscle regeneration of adult and old muscle *in vivo*. Interestingly, we found significantly higher numbers of CNFs within 5 days after injury in adult BI4500-treated animals (**Figure 5A, 6A**). This suggests there is a role for mitochondrial ROS production in inhibiting the formation of centrally nucleated fibers. The increase in CNFs in adult BI4500-treated animals contributed to a full recovery in mean fiber CSA at 35 days post injury. However, this did not translate to a difference in tissue fibrosis or force recovery at 35 days post-injury.

Generally, there were no differences between old placebo and old BI treated animals for any metric of muscle regeneration except force recovery. Increased CNFs and CSA recovery observed in adult BI-treated animals were not observed in old BI-treated animals. These results do not support the hypothesis that inhibition of RET O_2_^−^ release from mitochondrial site I_Q_ would improve muscle recovery in aged muscle. Based on the working hypothesis that the level of induction for CI ROS tightly regulates muscle differentiation, it is possible that no effect of BI4500 treatment was observed in old muscle due to the lower age-related capacity we observed to produce ROS from site I_Q_ caused by lower CI-linked respiratory capacities (**Figure 2, 3B-C, 4A**).

*Limitations*

We initially opted for chow delivery of the BI4500 to minimize the handling of the mice in this study. Despite the significant effects of this delivery on the target the plasma levels of the compound were below the level of quantitation and the single value for muscle compound concentration was below the target level. Because of this we changed to delivery by oral gavage for the muscle injury aspect of this study to achieve a higher peak circulating compound content. The once a day bolus dose instead of the more spread out dose available from chow feeding and the daily handling and stress that accompanies oral gavage could confound these results and limit our conclusions to oral gavage delivery method.

It is unclear why we did not see an increase in Pax7 positive nuclei in injured muscle at 5 days after injury given previous reports showing an increase in satellite cell number after injury[38]. One potential explanation is the timeline of the recovery relative to the severity of the injury. The BaCl_2_ injury appears very severe in H&E cross sections, with very little muscle tissue present. Others using cardiotoxin as an injury model have shown less severe pathology at a similar timeframe of recovery[39]. The severity of the injury could be affecting the timeline of recovery, which could explain the lack of differences in Pax7 positive cells between injured limbs.

*Conclusions.* We have demonstrated that BI4500 is a specific inhibitor of complex I_Q_ RET. CI RET is implicated in multiple disease and pathological conditions including ischemia-reperfusion. This compound provides another tool to dissect the importance of site-specific mitochondrial ROS production and may have potential as a therapeutic approach for preventing pathology associated with ischemia-reperfusion injury.

**Acknowledgements**

We would like to thank the *in vivo* services team at University of Washington for their help and support in the oral gavage studies. The small molecule BI4500 Complex I ROS inhibitor was obtained after having participated and being selected as a winner of a Molecule for Collaboration call launched on the  Boehringer Ingelheim’s open innovation platform OpnMe ([https://opnme.com](https://eur03.safelinks.protection.outlook.com/?url=https%3A%2F%2Fopnme.com%2F&data=05%7C01%7Cantonio.filareto%40boehringer-ingelheim.com%7Ce7fd7ba9d1444d6abcc308da91b959fd%7Ce1f8af86ee954718bd0d375b37366c83%7C0%7C0%7C637982522502197744%7CUnknown%7CTWFpbGZsb3d8eyJWIjoiMC4wLjAwMDAiLCJQIjoiV2luMzIiLCJBTiI6Ik1haWwiLCJXVCI6Mn0%3D%7C3000%7C%7C%7C&sdata=8UTyobb2eIxKXWNNgIUA87e%2B7x%2BQyKq1oKSjFNSRLmc%3D&reserved=0)).

**Funding**

Funding was provided by Boehringer Ingelheim and the National Institute on Aging (NIA) grants (T32AG066574, F32AG074655, P01AG001751).

**Conflict of Interest**

Antoni Filareto, Jens Markus Borghardt, and Michael Franti are employees of Boehringer Ingelheim. All other authors declare that they have no commercial or financial conflicts of interest. The authors worked with Boehringer Ingelheim on the design of this study. Otherwise, the funders played no role in the decision to publish this manuscript.

**Figure Legends**.

**Graphical Abstract.** BI4500 inhibits superoxide (O_2_^-^) production from the ubiquinone (Q)-binding site of complex I (I_Q_) resulting in decreases complex I reactive oxygen species (ROS) production and maintained complex I oxygen consumption rate (OCR). Optimal release of ROS from Complex I_Q_ signals to induce muscle differentiation. Treatment with BI4500 increased formation of centrally nucleated fibers (CNFs) and improves muscle fiber cross-sectional area (CSA) regeneration in injured muscle fibers of adult but not old mice. Sites of superoxide release: Complex I flavin binding site (I_F_), Complex II flavin binding site (II_F_), Complex III ubiquinone binding site (III_Q_). Protons (H^+^) and electrons (e^-^).

**Figure 1.** A) I_Q_ ROS dose response: Gastrocnemius (gastroc), quadriceps femoris (quad), and tibialis anterior (TA) muscles were dissected from adult 12 mo and old 24-26 mo mice, their mitochondria were isolated, and then ROS production from site I_Q_ was measured with a dose response of BI45000.

B) CI-Linked respiration and ROS: the gastrocnemius (gastroc) muscle was dissected from adult 6 mo or old 29 mo female mice and fibers were mechanically separated, permeabilized, and washed. During the permeabilization and wash steps, fibers were exposed to vehicle or BI4500. CI-linked respiration and ROS production was measured.

C) Chow feeding study: Adult 9-10 mo and old 22-24 mo female mice were fed control chow or BI4500 chow (580 mg/kg) for 8 weeks. Mitochondria were isolated from their muscle and CI site-specific ROS production was measured. Plasma BI4500 concentration was determined.

D) Oral Gavage PK study: 4 mo male mice were given daily oral gavage of BI4500 (30 mg BI4500/kg body mass) for 7 days. Plasma BI4500 concentration was determined after 7 days.

E) Muscle regeneration study design: Adult and aged animals were treated with BI4500 daily for 5 days or 35 days after muscle injury.

**Figure 2. BI4500 Decreases ROS Release from Site I_Q_ in a Concentration-Dependent Manner from Isolated Muscle Mitochondria.** Site I_Q_ ROS with an *ex vivo* BI4500 dose response in isolated muscle mitochondria from adult (12 mo) and old (24-26 mo) female mice (n=8). Mean ± SD.

**Figure 3. BI4500 Decreases ROS Release Without Impairing Respiration in Permeabilized Muscle Fibers.** A) ROS production with succinate (left), I_Q_ reverse (left middle), I_F_ reverse (right middle), and basal (right) conditions, B) ROS produced per O_2_ consumed with succinate, and C) OCR capacities with CI (left), CI&CII (middle), and CII-linked substrates (right). Adult (Yng; 6 mo, n=6-8) or old (29 mo, n=6) permeabilized gastrocnemius fibers from female mice. Two-way ANOVA with Sidak’s post hoc except point comparison of I_Q_ reverse old by unpaired two-tailed student’s t-test. *p<0.05. ns – not significant. Mean ± SD.

**Figure 4. BI4500 Chow Feeding Decreases Superoxide Release from Site I_Q_ *In Vivo*.** Site-specific ROS production for A) I_Q_ reverse (left), I_F_ reverse (middle), I_F_ forward (right), and B) Total CI reverse in adult (12 mo, n=8 control, n=5 8 weeks BI chow) or old (24-26 mo, n=8 control, n=4 8 weeks BI chow) isolated muscle mitochondria from female mice. For the aged mice, comparison of C) Body mass and D) Muscle masses normalized to body mass for the quadriceps femoris (left), gastrocnemius (middle), and tibialis anterior (right) muscles. *p<0.05, **p<0.01, ***p<0.001, ****p<0.0001. ns – not significant. Mean ± SD.

**Figure 5. Muscle regeneration five days after injury.** A) Within animal paired comparisons of % CNFs (left), percent difference of CNFs (center; ∆ = BaCl_2_ – Sham), and representative images (right) of H&E-stained TA cross sections from BaCl_2_ injured and sham muscles five days after muscle injury. B) Within animal pairwise comparisons of muscle fibrosis (left), percent difference in fibrosis (center), and representative images (right) of Sirius red stained TA cross sections from BaCl_2_ injured and sham muscles five days after muscle injury. C) Within animal pairwise comparisons of Pax7 positive nuclei (left), percent difference in Pax7 positive nuclei (center), and representative images (right) of Pax7 stained TA cross sections from BaCl_2_ injured and sham muscles five days after muscle injury. Pax7 (red) was used as a marker of satellite cells, Laminin (green), and DAPI (blue). Nuclei were counted as satellite cells if DAPI and Pax7 overlapped (magenta), illustrated by the white arrows. *p<0.05, **p<0.01, ***p<0.001, ****p<0.0001. Mean ± SEM. All samples from adult (9-11 mo) and old (27 mo) male mice.

**Figure 6. Muscle Centrally Nucleated Fibers and Cross-Sectional Area 35 days after injury.** A) Within animal paired comparisons of % CNFs (left), percent difference of CNFs (center; ∆ = BaCl_2_ – Sham), and representative images (right) of H&E-stained TA cross sections from BaCl_2_ injured and sham muscles from contralateral hindlimbs 35 days after muscle injury. B) Within animal pairwise comparisons of CSA (left) and difference of mean CSA between injured and sham limbs (right;). C) Fiber CSA distribution of sham limbs (left) and BaCl_2_ injured limbs (right). *p<0.05, **p<0.01, ***p<0.001, ****p<0.0001, ns – not significant. Mean ± SEM. All samples from adult (9-11 mo) and old (27 mo) male mice.

**Figure 7. Muscle fibrosis and satellite cell abundance 35 days after injury.** A) Within animal pairwise comparisons of muscle fibrosis (left), percent difference in fibrosis (center; ∆ = BaCl_2_ – Sham), and representative images (right) of Sirius red stained TA cross sections from BaCl_2_ injured and sham muscles from contralateral hindlimbs 35 days after muscle injury. B) Within animal pairwise comparisons of Pax7 positive nuclei (left), percent difference in Pax7 positive nuclei (center), and representative images (right) of Pax7/Laminin/DAPI (Red/Green/Blue) stained TA cross sections from BaCl_2_ injured and sham muscles 35 days after muscle injury. White arrows indicate Pax7 positive nuclei. *p<0.05, **p<0.01, ***p<0.001. Mean ± SEM. All samples from adult (9-11 mo) and old (27 mo) male mice.

**Figure 8. Maximal TA force recovery is not different between ages or treatment groups 35 days after injury.** A) Within animal pairwise comparisons of maximal TA muscle force in sham vs injured limbs stratified by age and treatment. B) Difference of maximal force output between injured and sham limbs (∆ = BaCl_2_ – Sham). *p<0.05 Mean ± SEM. All samples from adult (9-11 mo) and old (27 mo) male mice.

REFERENCES

1. Hoppeler, H., *Molecular networks in skeletal muscle plasticity.* J Exp Biol, 2016. **219**(Pt 2): p. 205-13.

2. Flück, M. and H. Hoppeler, *Molecular basis of skeletal muscle plasticity--from gene to form and function.* Rev Physiol Biochem Pharmacol, 2003. **146**: p. 159-216.

3. Slivka, D., et al., *Single muscle fiber adaptations to resistance training in old (>80 yr) men: evidence for limited skeletal muscle plasticity.* Am J Physiol Regul Integr Comp Physiol, 2008. **295**(1): p. R273-80.

4. Degens, H. and S.E. Alway, *Skeletal muscle function and hypertrophy are diminished in old age.* Muscle Nerve, 2003. **27**(3): p. 339-47.

5. Baker, B.A., et al., *Adaptive stretch-shortening contractions: diminished regenerative capacity with aging.* Appl Physiol Nutr Metab, 2008. **33**(6): p. 1181-91.

6. Li, R., et al., *Associations of Muscle Mass and Strength with All-Cause Mortality among US Older Adults.* Medicine and science in sports and exercise, 2018. **50**(3): p. 458-467.

7. Sousa-Victor, P., L. García-Prat, and P. Muñoz-Cánoves, *Control of satellite cell function in muscle regeneration and its disruption in ageing.* Nat Rev Mol Cell Biol, 2022. **23**(3): p. 204-226.

8. Qaisar, R., S. Bhaskaran, and H. Van Remmen, *Muscle fiber type diversification during exercise and regeneration.* Free Radic Biol Med, 2016. **98**: p. 56-67.

9. Parker, M.H., *The altered fate of aging satellite cells is determined by signaling and epigenetic changes.* Front Genet, 2015. **6**: p. 59.

10. Bhattacharya, D. and A. Scimè, *Mitochondrial Function in Muscle Stem Cell Fates.* Front Cell Dev Biol, 2020. **8**: p. 480.

11. Le Moal, E., et al., *Redox Control of Skeletal Muscle Regeneration.* Antioxid Redox Signal, 2017. **27**(5): p. 276-310.

12. Campbell, M.D., et al., *Improving mitochondrial function with SS-31 reverses age-related redox stress and improves exercise tolerance in aged mice.* Free Radic Biol Med, 2019. **134**: p. 268-281.

13. Siegel, M.P., et al., *Mitochondrial-targeted peptide rapidly improves mitochondrial energetics and skeletal muscle performance in aged mice.* Aging cell, 2013. **12**(5): p. 763-771.

14. Basisty, N., et al., *Mitochondrial-targeted catalase is good for the old mouse proteome, but not for the young: ‘reverse’ antagonistic pleiotropy?* Aging Cell, 2016. **15**(4): p. 634-645.

15. Kruse, S.E., et al., *Age modifies respiratory complex I and protein homeostasis in a muscle type-specific manner.* Aging Cell, 2016. **15**(1): p. 89-99.

16. Wong, H.S., et al., *Production of superoxide and hydrogen peroxide from specific mitochondrial sites under different bioenergetic conditions.* J Biol Chem, 2017. **292**(41): p. 16804-16809.

17. Chouchani, E.T., et al., *Ischaemic accumulation of succinate controls reperfusion injury through mitochondrial ROS.* Nature, 2014. **515**(7527): p. 431-435.

18. Brand, M.D., et al., *Suppressors of Superoxide-H 2 O 2 Production at Site I Q of Mitochondrial Complex I Protect against Stem Cell Hyperplasia and Ischemia-Reperfusion Injury.* Cell Metab, 2016. **24**(4): p. 582-592.

19. Rimal, S., et al., *Reverse electron transfer is activated during aging and contributes to aging and age‐related disease.* EMBO reports, 2023.

20. Malinska, D., et al., *Changes in mitochondrial reactive oxygen species synthesis during differentiation of skeletal muscle cells.* Mitochondrion, 2012. **12**(1): p. 144-8.

21. Schieber, M. and N.S. Chandel, *ROS function in redox signaling and oxidative stress.* Curr Biol, 2014. **24**(10): p. R453-62.

22. Hom, J.R., et al., *The permeability transition pore controls cardiac mitochondrial maturation and myocyte differentiation.* Dev Cell, 2011. **21**(3): p. 469-78.

23. Lee, S., et al., *Mitochondrial H2O2 generated from electron transport chain complex I stimulates muscle differentiation.* Cell Res, 2011. **21**(5): p. 817-34.

24. Wong, H.S., P.A. Monternier, and M.D. Brand, *S1QELs suppress mitochondrial superoxide/hydrogen peroxide production from site I Q without inhibiting reverse electron flow through Complex I.* Free Radic Biol Med, 2019. **143**: p. 545-559.

25. Quinlan, C.L., et al., *The determination and analysis of site-specific rates of mitochondrial reactive oxygen species production.* Methods Enzymol, 2013. **526**: p. 189-217.

26. Quinlan, C.L., et al., *Sites of reactive oxygen species generation by mitochondria oxidizing different substrates.* Redox Biol, 2013. **1**: p. 304-12.

27. Pharaoh, G., et al., *Reduced adenosine diphosphate sensitivity in skeletal muscle mitochondria increases reactive oxygen species production in mouse models of aging and oxidative stress but not denervation.* JCSM Rapid Communications, 2021. **4**(1): p. 75-89.

28. Ahn, B., et al., *Nrf2 deficiency exacerbates age-related contractile dysfunction and loss of skeletal muscle mass.* Redox Biol, 2018. **17**: p. 47-58.

29. Kuznetsov, A.V., et al., *Analysis of mitochondrial function in situ in permeabilized muscle fibers, tissues and cells.* Nat Protoc, 2008. **3**(6): p. 965-76.

30. Wang, C., F. Yue, and S. Kuang, *Muscle Histology Characterization Using H&E Staining and Muscle Fiber Type Classification Using Immunofluorescence Staining.* Bio Protoc, 2017. **7**(10).

31. Pharaoh, G., et al., *Targeting cPLA 2 derived lipid hydroperoxides as a potential intervention for sarcopenia.* Scientific Reports, 2020. **10**(1): p. 1-18.

32. Wang, Y., et al., *Increases of M2a macrophages and fibrosis in aging muscle are influenced by bone marrow aging and negatively regulated by muscle-derived nitric oxide.* Aging Cell, 2015. **14**(4): p. 678-88.

33. Alnaqeeb, M.A., N.S. Al Zaid, and G. Goldspink, *Connective tissue changes and physical properties of developing and ageing skeletal muscle.* J Anat, 1984. **139 ( Pt 4)**: p. 677-89.

34. Banba, A., et al., *Defining the mechanism of action of S1QELs, specific suppressors of superoxide production in the quinone-reaction site in mitochondrial complex I.* J Biol Chem, 2019. **294**(16): p. 6550-6561.

35. Treberg, J.R., C.L. Quinlan, and M.D. Brand, *Evidence for two sites of superoxide production by mitochondrial NADH-ubiquinone oxidoreductase (complex I).* J Biol Chem, 2011. **286**(31): p. 27103-10.

36. Rodríguez-Nuevo, A., et al., *Oocytes maintain ROS-free mitochondrial metabolism by suppressing complex I.* Nature, 2022. **607**(7920): p. 756-761.

37. Tierney, M.T., et al., *Muscle Stem Cells Exhibit Distinct Clonal Dynamics in Response to Tissue Repair and Homeostatic Aging.* Cell Stem Cell, 2018. **22**(1): p. 119-127 e3.

38. Conboy, I.M., et al., *Rejuvenation of aged progenitor cells by exposure to a young systemic environment.* Nature, 2005. **433**(7027): p. 760-4.

39. Cardoso, E.S., et al., *Thymol accelerates the recovery of the skeletal muscle of mice injured with cardiotoxin.* J Pharm Pharmacol, 2016. **68**(3): p. 352-60.
