# Supplementary material for: A novel mitochondrial complex I ROS inhibitor partially improves muscle regeneration in adult but not old mice
Source: Redox Biol. 2023 Jun 2;64:102770. doi: 10.1016/j.redox.2023.102770 (PMC10267642; doi:10.1016/j.redox.2023.102770)
Supplement: Multimedia component 2 [file mmc2.docx]

| **Site** | **Substrates** | **Inhibitor Control** |
| --- | --- | --- |
| I_F_ Reverse | 5 mM succinate, 1 μM nigericin, and 0.3 μM S3QEL1.2 | 0.3 µM S3QEL1.2 + 1 µM nigericin |
| I_Q_ Reverse | 5 mM succinate and 0.3 μM S3QEL1.2 | 5 mM Succinate + 0.3 µM S3QEL1.2 + 1 µM nigericin |
| O_F_ Forward | 5 mM glutamate, 5 mM malate, 4 µM rotenone | 4 µM rotenone |
